# Sphingosine-1-phosphate receptor signaling regulates an ERK1/2–p65 molecular switch in macrophages during *Leishmania donovani* infection

**DOI:** 10.3389/fphar.2026.1769137

**Published:** 2026-02-10

**Authors:** Mohd Arish, Farha Naz

**Affiliations:** 1 JH-Institute of Molecular Medicine, Jamia Hamdard, New Delhi, India; 2 Division of Infectious Disease and International Health, Department of Medicine, University of Virginia, Charlottesville, VA, United States

**Keywords:** ERK (extracellular signal-regulated kinase), *L. donovani*, macrophages, Nf-κb (nuclear factor-kappa b), sphingosine-1-phosphate signaling

## Abstract

*Leishmania donovani* establishes intracellular infection by suppressing macrophage inflammatory responses. Although sphingosine-1-phosphate (S1P) signaling is known to regulate macrophage function, the receptor isotype–specific mechanisms involved during *Leishmania donovani* infection remain unclear. In this study, we examined the role of individual S1P receptors (S1PR1–3) in modulating macrophage responses to *L. donovani.* Using *L. donovani–*infected THP-1–derived human macrophages, selective pharmacological agonists of S1PR1, S1PR2, and S1PR3 were employed to assess intracellular signaling, inflammatory mediator production, and parasite burden. Activation of S1PR signaling differentially regulated an ERK1/2–NF-κB p65 molecular switch, marked by reduced ERK1/2 phosphorylation and enhanced p65 phosphorylation. These changes were associated with decreased IL-10 levels, increased TNF-α and nitric oxide production, and reduced intracellular parasite load. While activation of all three receptor isotypes limited parasite survival, S1PR2 produced the most pronounced inflammatory and anti-leishmanial effects, indicating functional divergence among S1PRs. These findings highlight receptor-specific roles of S1PR signaling in macrophage responses during *L. donovani* infection.

## Introduction

Macrophages are highly plastic cells of the immune system that offer protection against various intracellular pathogens. However, intracellular pathogens exploit macrophages by taking advantage of macrophage plasticity, resulting in disease progression (Arish and Naz, 2022a). *Leishmania donovani* (AG83) strain, causative agent for Visceral Leishmaniasis, is a protozoan parasite that can infect macrophages (Lang et al., 1994). BALB/c mice are highly susceptible to *Leishmania* infection, as they fail to polarize macrophages toward a protective M1 phenotype, instead favoring a Th2-driven environment that allows the parasite to persist (Bodhale et al., 2018). Diseased susceptible macrophages are often characterized by anti-inflammatory phenotypes, while host-protective phenotypes are associated with inflammatory responses such as increased inducible nitric oxide (iNOS), Interleukin (IL)-12, and Tumor necrosis factor (TNF)-α production (Carneiro et al., 2021). Hence, macrophage reprograming from anti-inflammatory to pro-inflammatory can be used as an adjunctive therapy for the management of Leishmaniasis (Palomino-Cano et al., 2024).

Sphingosine-1-phosphate (S1P) signaling has been reviewed as an important drug target against several infectious diseases (Arish et al., 2016; Naz et al., 2024). Previously, we studied the role of S1P signaling in clearing intracellular *L. donovani* and *Mycobacteria tuberculosis* infection through activation of S1P signaling. This leads to macrophages activation to secret pro-inflammatory mediators such as IL-12 or IL-6 that further leads to infection clearance (Arish et al., 2018; Arish and Naz, 2022b). As S1P signaling could be mediated through its five isotypes, named as S1P receptor (S1PR) 1–5 (Arish et al., 2017; McGinley and Cohen, 2021). Monocytes and macrophages express multiple S1PRs primarily S1PR1, S1PR2, and S1PR3 and is crucial for their migration, recruitment to inflammation sites, phagocytosis, and inflammatory responses, where S1PR1 often promotes anti-inflammatory effects and S1PR2/3 drive pro-inflammatory actions like pro-IL1β, IL-18, and TNF-α,release (Lin et al., 2024; Wang et al., 2023; Weigert et al., 2019). Although S1PR4/5 expression is found in low levels in macrophages, its role in regulation of macrophage function is largely unknow (Weigert et al., 2019). THP-1 derived macrophages only express S1PR1-3 and play important role in macrophage polarization following Mtb infection (Arish and Naz, 2022b). Nevertheless, the role of S1P signaling was studied previously (Arish, et al., 2018), receptor specific anti-leishmanial responses and underlying molecular mechanisms in context of *L. donovani* infection was not well understood.

In the present study, we further elucidate the role of S1P signaling in regulating the extracellular signal-regulated kinase (ERK) and nuclear factor kappa B (NF-κB) pathways during *L. donovani* infection. As S1P is a natural ligand of S1PRs, we used specific S1PR1-3 agonists, named as CYM5442, CYM5520, and CYM5541, respectively, to further examine the exact receptor-mediated anti-leishmanial response. CYM5442 is a potent and selective agonist of S1PR1 (Gonzalez-Cabrera et al., 2008), CYM5520 selectively activates S1PR2 without cross-reactivity to other (Satsu et al., 2013), and CYM5541 exhibits preferential activation of S1PR3 (Jo et al., 2012). In this study we looked for ERK1/2 and p65 activation status in S1PR1-3-agonist treated *L. donovani*-infected THP-1 derived macrophages. As S1P signaling have previously shown induction of pro-inflammatory functions of the macrophages (Tian et al., 2023; Yang et al., 2018), we next assessed the capacity of S1PR1-3 in eliminating intracellular *L. donovani* infection.

## Methods

### Human macrophage cell line

The THP-1 cell line was maintained in RPMI 1640 medium (Life Technologies) supplemented with 10% heat-inactivated FBS (Life Technologies) and 100 U/mL penicillin and 100 μg/mL streptomycin formulation (Life Technologies) at 37 °C in 5% CO_2_. PMA (phorbol 12-myristate 13-acetate; Sigma) was used for differentiation of THP-1 monocytes into macrophages by incubating THP-1 monocytes for 24 h with 5 ng/mL PMA, followed by 24 h resting at 37 °C in 5% CO_2_ in flat-bottom 12-well tissue culture plates (BD Biosciences). To preserve the optimal phenotype of THP-1 monocytes, the cell line was not passaged more than 20 times.

### Parasite

The standard strain of *L. donovani*: (MHOM/IN/83/AG83) was maintained in M199 media (Life Technologies) with 25 mM HEPES (Sigma) and supplemented with 10% heat-inactivated Fetal bovine serum (Life Technologies) with 100 U/mL penicillin and 100 μg/mL streptomycin formulation (Life Technologies) at 22 °C. Mid-log phase culture was used to infect differentiated THP-1 cells. *Leishmania donovani* strain used in this study was routinely passaged through mouse infection and periodically revived from low-passage frozen stocks.

### Infection of macrophages

THP-1 derived macrophages were harvested and distributed into 12-well plates at 1 × 10^6^/mL. Promastigotes were added to differentiated macrophages at an infection ratio of 1:10 for 6 h to initiate infection. Infected macrophages were further replenished with supplemented RPMI 1640 containing 10% FBS for an additional 24 h for different studies.

### Parasite load

Macrophages were seeded on poly-L-lysine–coated sterile coverslips placed in 12-well culture plates at a density of 1 × 10^6^ cells/mL. Cells were infected with *L. donovani* at a 1:10 ratio for 6 h, after which extracellular parasites were gently removed by washing. Infected cells were treated with 10 μM S1PR1-3 agonist and cultured for an additional 24 h.

Following incubation, cells were washed with PBS, fixed with ice-cold methanol for 5 min, and air-dried. Coverslips were then immersed in Giemsa stain for 30 min and washed 2–3 times with PBS. For each condition, at least 15 randomly selected fields were examined microscopically to determine the average number of parasites per macrophage. Parasite load for each treatment was expressed as a percentage relative to the control group, which was set at 100%.

### Protein extraction and western blotting

Following experimental treatments, infected and uninfected THP-1–derived macrophages were washed twice with ice-cold phosphate-buffered saline (PBS) and lysed using ice-cold lysis buffer (Cell Signaling Technology) supplemented with 1× protease inhibitor cocktail and 1× phosphatase inhibitor cocktail (Sigma). Cell lysates were clarified by centrifugation at 14,000 × g for 15 min at 4 °C, and the supernatants were collected.

Protein concentrations were determined using a commercial protein quantification assay according to the manufacturer’s instructions (Bradford assay, Bio-Rad). Equal amounts of protein (approximately 80 µg per lane) were mixed with SDS sample buffer (ThermoScientific), denatured by heating, and resolved by SDS–PAGE on 12% polyacrylamide gels using commercially available running buffer. Proteins were subsequently transferred onto polyvinylidene difluoride (PVDF) membranes using a commercially available transfer buffer supplemented with 20% methanol.

Following transfer, membranes were blocked in blocking buffer and incubated with primary antibodies against phospho-ERK1/2, total ERK1/2, phospho-NF-κB p65, total p65, and β-actin (Cell Signaling Technology). After washing with wash buffer, membranes were incubated with appropriate horseradish peroxidase–conjugated secondary antibodies. Immunoreactive bands were detected using a chemiluminescence-based detection system according to the manufacturer’s instructions. Band intensities were quantified by densitometric analysis using ImageJ software and normalized to β-actin or the corresponding total protein levels, as indicated.

### Enzyme-linked immunosorbent assay

Cytokine levels in culture supernatants from *L. donovani-*infected and uninfected THP-1-derived macrophages with or without S1PR1-3 agonist were quantified using BD Biosciences ELISA kits, following the manufacturer’s protocol with minor optimizations. Briefly, supernatants were collected at indicated time points, centrifuged at 300 × *g* for 5 min to remove cellular debris, and stored at −80 °C until analysis. Ninety-six-well plates were coated with capture antibody (100 µL/well) and incubated overnight at 4 °C. After washing with PBS containing 0.05% Tween-20, wells were blocked with assay diluent for 1 h at room temperature. Samples and serially diluted standards were added in duplicate (100 µL/well) and incubated for 2 h at room temperature. Plates were washed three times and incubated with biotin-conjugated detection antibody (100 µL/well) for 1 h, followed by streptavidin-HRP (100 µL/well) for 30 min in the dark. TMB substrate (100 µL/well) was added, and the reaction was stopped with 50 µL stop solution. Absorbance was measured at 450 nm with a 570 nm reference using a microplate reader. Cytokine concentrations were determined from standard curves using four-parameter logistic regression.

### Nitric oxide assay

Nitric oxide (NO) production was measured using the Nitric Oxide Assay Kit (Thermo Scientific), which quantifies total nitrite as an indicator of NO. Cell-free supernatants were mixed with equal volumes of Griess reagent (50 µL + 50 µL) in 96-well plates and incubated at room temperature for 10 min in the dark. Nitrite standards (0–100 µM) were prepared in culture medium to account for background. Absorbance was recorded at 540 nm, and nitrite concentrations were interpolated from the standard curve. All measurements were performed in triplicate and expressed as µM nitrite per mL of culture supernatant.

### Statistical analysis

The statistical analysis was performed using GraphPad Prism, version 7.0 (GraphPad, San Diego, CA, USA). To ascertain the significance of the difference between the means of two samples Student’s t-test was used. One-way ANOVA with Tukey test was performed for multiple comparisons. The error bars of the values represent ± SD from the replicates. The results shown are a representation from a minimum of three similar experiments that generated reproducible data.

## Results

### Reciprocal regulation of pro and anti-inflammatory profiles in macrophages by S1PR1-3 agonists

**FIGURE 1:**
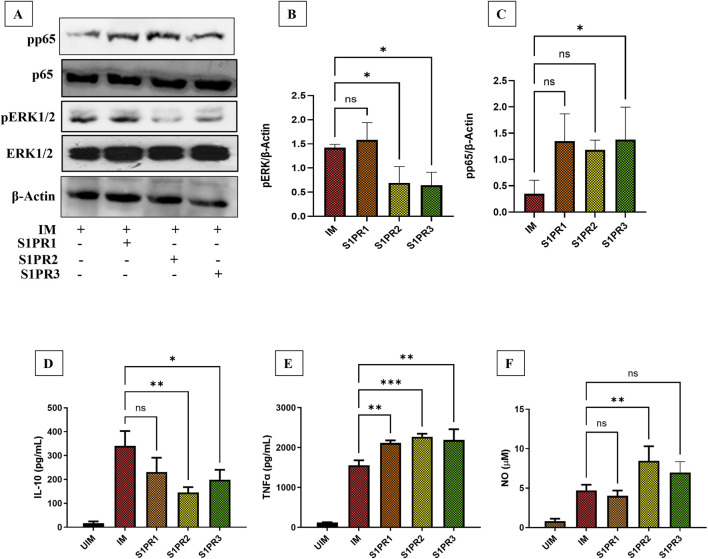
Reciprocal regulation of pro and anti-inflammatory profiles in macrophages by S1PR1-3 agonists THP-1 derived macrophages were cultured in six-well plates in the presence of *L. donovani* infection (MOI = 1:10) for 6 h, infected macrophages (IM) were washed to remove non-internalized parasites and incubated for next 24 h in presence or absence of 10μM S1PR1, S1PR2, and S1PR3 agonist, respectively **(A)** Western Blot analysis showing p65 and ERK1/2 phosphorylation. **(B)** Densitometry analysis of pERK western blot **(C)** Densitometry analysis of p65 western blot **(D)** IL-10 production in *L. donovani* infected and uninfected THP-1 derived macrophages (UIM) supplemented with S1PR1-3 agonists for additional 24 h **(E)** TNFα production in *L. donovani* infected and uninfected THP-1 derived macrophages supplemented with S1PR1-3 agonists for additional 24 h **(F)** NO production in *L. donovani* infected and uninfected THP-1 derived macrophages supplemented with S1PR1-3 agonists for additional 24 h. The data is a representation of mean ± SD from three independent experiments *, p< 0.05; **, p< 0.01; ***, p < 0.001;ns non-significant.

*Leishmania donovani* infection has been previously shown to suppress macrophage-mediated inflammatory responses to facilitate its intracellular persistence (Gupta et al., 2016). It is previously known that *L. donovani* modulates ERK and NF-κB pathways (Reinhard et al., 2012; Yang et al., 2007). To determine whether S1PR modulators can modulate ERK signaling and NF-κB pathway, we treated *L. donovani*–infected THP-1–derived macrophages with selective agonists of S1PR1–3 (10 μM). As THP-1–derived macrophages express S1PR1–3 (Arish et al., 2018) only these receptor agonists were used in this study. We observed that activation of S1PR2 and S1PR3 significantly reduced ERK phosphorylation in infected macrophages (Figures 1A,B).

NF-κB activation is required for protection against *L. donovani* infection (Reinhard et al., 2012). To assess NF-κB activation, we next analyzed phospho-p65 (Rel/A) levels following S1PR1–3 agonist treatment. Notably, S1PR1–2 agonists induced non-significant increase in NF-κB activation in infected THP-1 macrophages. However, S1PR3 agonist showed significant increase expression of pp65 (Figures 1A,C). As ERK phosphorylation leads to IL-10 production (Yang et al., 2007), we checked if this decrease in phospho-ERK level leads to low IL-10 production in infected macrophages after activation of S1P signaling. We observed that after S1PR1-3 agonist supplementation, there was low level of IL-10 detected in the supernatant in the infected macrophages (Figure 1D). Given our previous observation that S1P signaling enhances TNFα production in *M. tuberculosis*–infected macrophages (Arish and Naz, 2022b), we investigated whether a similar effect occurs in *L. donovani* infection. Consistent with this, TNFα production was significantly elevated in infected macrophages following S1P pathway activation (Figure 1E).

Finally, we assessed NO production, as NF-κB activity has been reported to regulate NO synthesis (Jones et al., 2007; Li et al., 2002). In agreement with our previous findings that S1PR1–3 modulators promote NO generation in THP-1 macrophages during *M. tuberculosis* infection (Arish and Naz, 2022b), we observed a significant increase in NO levels specifically in the S1PR2 agonist–treated group, with non-significant increase following S1PR3 agonist treatment as compared to infected macrophages (Figure 1F).

### S1PR1-3 mediated signaling results in a decrease in parasite load

**FIGURE 2:**
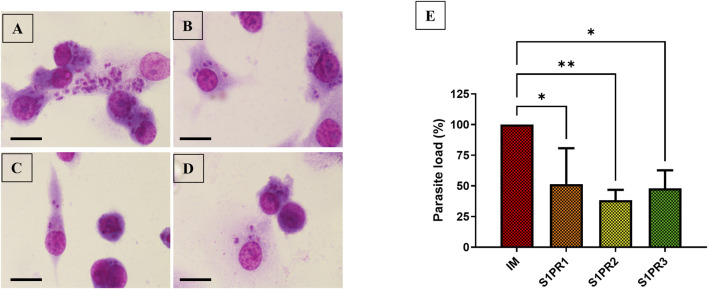
S1PR1-3 mediated signaling decreases parasite load: THP-1 derived macrophages were cultured on a cover-slip in six-well plates in the presence of *L. donovani* infection (MOI = 1:10) for 6 h, macrophages were washed to remove non-internalized parasites and incubated for next 24 h in presence or absence of 10 μM S1PR1, S1PR2, and S1PR3 agonist. **(A)** IM **(B)** S1PR1 agonist-treated infected macrophages **(C)** S1PR2 agonist-treated infected macrophages **(D)** S1PR3 agonist-treated infected macrophages **(E)** Graphical representation of percentage parasite load in infected and S1PR1-3-treated infected macrophages. Scale bar 10 μm. The data is a representation of mean ± SD from three independent experiments *, p< 0.05; **, p< 0.01; ns non-significant.

Our previous work demonstrated that S1P mediates anti-leishmanial activity in macrophages through reciprocal regulation of IL-10 and IL-12 (Arish et al., 2018). Additionally, we have further shown that S1PR2-3 signaling promotes M1 phenotype in THP-1 derived resting macrophages, which further reduces intracellular *M. tuberculosis* infection (Arish and Naz, 2022b). To further delineate the contribution of individual receptors, we employed selective agonists for S1PR1–3. We found that all three receptors exerted anti-leishmanial effects to varying degrees (Figures 2A–E), with the S1PR2 agonist showing the most pronounced and statistically significant reduction in parasite load compared to control, S1PR1 or S1PR3-agonist treated THP-1–derived infected macrophages (Figures 2A–E).

## Discussion

The hallmark of successful *L. donovani* infection is marked by suppression of protective pro-inflammatory immune responses together with enhanced anti-inflammatory signaling, which collectively favor parasite survival within host macrophages (da Silva et al., 2021). The later phenotypic changes are determined by key biomarkers of these responses, such as ERK-mediated IL-10 production that suppresses pro-inflammatory responses. *Leishmania donovani* strongly induces ERK1/2 phosphorylation in macrophages, which is further contributes to IL-10 production (Yang et al., 2007). In addition, IL-10 negatively regulates inflammatory cytokines such as IL-12 and TNFα (Kane and Mosser, 2001). Mice lacking the IL-10 gene have less parasitemia, and treatment of infected macrophages with recombinant IL-10 results in increased parasite load (Kane and Mosser, 2001). In our study, we found decreased phosphorylation in ERK1/2 and increased IL-10 levels in the *L. donovani*-infected macrophages, however, treatment of THP-1 derived macrophages with S1PR2-3 resulted in decreased ERK1/2 phosphorylation and decreased IL-10 production. However, S1PR1 agonist treatment does not alter ERK1/2 phosphorylation, also there was non-significant decrease in IL-10 levels in macrophages suggesting that S1PR1 is not regulating ERK1/2 pathway in *L. donovani* infected macrophages.

NF-kB family of transcription factors has been previously shown to regulate inflammatory responses such as TNFα and NO production (Dorrington and Fraser, 2019; Xie et al., 1994), which is critical for host-protection. P65 phosphorylation is necessary for NF-kB activation (Kwon et al., 2016), and it was showed that *L. donovani* reprograms the host epigenome, which in turn inhibits NF-kB activation and thus results in the absence of inflammatory response, which allows the parasite to thrive inside macrophages (Lecoeur et al., 2020). Activation of iNOS and proinflammatory cytokine production through NF-kB was observed in fucoidan, an immunomodulator obtained from *Fucus vesiculosus*, treated infected macrophages, which leads to suppression of intracellular parasite growth (Sharma et al., 2014). In this context, it could be speculated that activation of NF-kB by immunomodulators could regain the ability of macrophages to fight against infection. Similarly, we found a significant phosphorylation of the p65 subunit of NF-kB upon treatment of all the S1PR1-3 agonists, whereas low phosphorylation of p65 was detected in *L. donovani*-infected macrophages. Interestingly, S1PR1 and S1PR3 agonists do not induce much NO production as compared to S1PR2 agonist, which may be attributed to lower IL-10 levels in macrophages treated with these agonists, as IL-10 is known to suppress NO production (Cunha et al., 1992). Taken together, macrophages treated with an S1PR2 agonist show increased NO levels, potentially due to reduced IL-10 and elevated TNFα levels. However, further investigation is required to confirm these findings in primary macrophages.

Finally, treatment with S1PR1–3 agonists led to a reduction in parasite load in infected macrophages, with the S1PR2 agonist producing the most pronounced clearance of intracellular parasites. This enhanced effect may be attributed to promote both NO secretion and TNFα production. While S1PR1 and S1PR3 activation also reduced parasite load, their effects are likely driven primarily by increased TNFα production. However, it is anticipated that some other mediators could be involved, as S1PR signaling can regulate macrophage polarization (Arish and Naz, 2022b). Although activation of all three S1PR isotypes reduced intracellular *L. donovani* burden, our data reveal clear receptor-specific differences in downstream inflammatory signaling. S1PR2 and S1PR3 activation markedly suppressed ERK1/2 phosphorylation and enhanced nitric oxide production, whereas S1PR1 exerted minimal effects on ERK1/2 signaling and NO generation. Despite this divergence, S1PR1 activation still significantly reduced parasite burden, indicating that parasite control can be achieved through distinct, non-redundant inflammatory pathways.

This differential behavior is consistent with established S1PR biology, wherein S1PR2 and S1PR3 preferentially engage signaling pathways that antagonize ERK-driven anti-inflammatory responses and promote NF-κB–dependent antimicrobial functions, including nitric oxide production (Yang et al., 2018; Arish and Naz, 2022b). In contrast, S1PR1 primarily signals through Gαi-dependent mechanisms and has been shown to modulate inflammatory responses independently of ERK1/2, potentially through TNF-α–biased NF-κB activation (Gonzalez-Cabrera et al., 2008; Tian et al., 2023). The convergence on parasite reduction despite divergence in signaling underscores that restoration of macrophage antimicrobial capacity does not require uniform activation of all inflammatory pathways. Rather, selective engagement of receptor-specific S1PR signaling modules may be sufficient to limit intracellular parasite survival, highlighting the therapeutic relevance of receptor-targeted S1PR modulation.

S1P receptor signaling has previously been shown to influence macrophage polarization in Mtb infection including the promotion of inflammatory phenotypes via S1PR2 and S1PR3 (Arish and Naz, 2022b). In the present study, however, macrophage polarization was not directly assessed using canonical M1 or M2 markers. Instead, functional and signaling readouts relevant to leishmaniasis were evaluated, including ERK1/2 and NF-κB p65 activation, IL-10 and TNF-α production, and NO generation, which are well-established determinants of parasite survival in *L. donovani–*infected macrophages.

Although macrophage polarization is critical for the elimination of infectious agents, an appropriately regulated inflammatory response is essential to prevent excessive tissue damage. While classically activated (M1) macrophages generate potent antimicrobial mediators such as nitric oxide and pro-inflammatory cytokines that aid in pathogen clearance, uncontrolled or prolonged activation can lead to collateral damage, chronic inflammation, and impaired tissue repair (Laskin et al., 2011). Conversely, alternatively activated (M2) macrophages contribute to the resolution of inflammation and tissue remodeling, but excessive skewing toward this phenotype may facilitate pathogen persistence and immune evasion. Therefore, a finely tuned balance between pro- and anti-inflammatory macrophage responses is fundamental to achieving effective host defense while preserving tissue integrity (Laskin et al., 2011). Taken together, our findings suggest that activation of S1PR1–3 contributes to the reduction of intracellular parasite burden by promoting inflammatory responses in human macrophages, as evidenced by increased nitric oxide and TNF-α production. However, the present study has certain limitations. First, these observations require validation in primary human and murine macrophages to more comprehensively define the role of individual S1PR agonists. Furthermore, *in vivo* studies will be necessary to delineate the specific contributions of distinct S1PRs in regulating macrophage responses during *L*. *donovani* infection.

## Data Availability

The raw data supporting the conclusions of this article will be made available by the authors, without undue reservation.
